# Usability assessment of seven HIV self-test devices conducted with lay-users in Johannesburg, South Africa

**DOI:** 10.1371/journal.pone.0227198

**Published:** 2020-01-14

**Authors:** Mohammed Majam, Laura Mazzola, Naleni Rhagnath, Samanta T. Lalla-Edward, Raees Mahomed, Willem Daniel Francois Venter, Alex Emilio Fischer

**Affiliations:** 1 Ezintsha, a sub-division of Wits Reproductive Health and HIV Institute, University of Witwatersrand, Johannesburg, Gauteng, South Africa; 2 Halteres Associates, San Francisco, California, United States of America; Boston University, UNITED STATES

## Abstract

**Introduction:**

The first 90 of the *90-90-90* initiative introduced by the World Health Organization(WHO) in 2015 requires 90% of people with HIV be aware of their status by 2020. In South Africa, conventional facility-based testing had reached 84.9% in 2018; innovative new methods, like HIV self-testing(HIVST) may close the testing gap. This study aimed to determine the usability of seven HIVST kits among untrained South Africans.

**Methods:**

This cross-sectional study of 1400 adults in Johannesburg evaluated the usability of five blood fingerstick and two oral fluid HIVSTs, using WHO prequalification criteria, from June 2016 to June 2018. Participants were handed one kit, with no further information about the device or test procedure, and asked to perform the test in front of an observer. The observer used product-specific semi-structured questionnaires organized into a composite usability index(UI) using a HIVST process checklist, a contrived results interpretation and a post-test interview that expanded on participant experiences with the device and instructions-of-use(IFU). Participants were not tested themselves, but provided with contrived results to interpret.

**Results:**

The average UI was 92.8%(84.2%-97.6%); the major difficulty was obtaining and transferring the specimen. Participants correctly interpreted 96.1% of the non-reactive/negative, 97.0% of the reactive/positive, 98.0% of the invalid and 79.9% of the weak positive results. Almost all participants(97.0%) stated they would visit a clinic or seek treatment for positive results; with negative results, half(50.6%) stated they should re-test in the next three months while one-third(36.1%) said they should condomize. Nearly all found the devices easy to use(96.6%), the IFUSs easy to understand(97.9%) and felt confident using the test unassisted(95.9%) but suggested improvements to packaging/IFUs to further increase usability; 19.9% preferred clinic-based testing to HIVST.

**Conclusion:**

The UI and interpretation of results was high and in-line with previous usability studies, suggesting that these kits are appropriate for use in the general, untrained and unsupervised public.

## Introduction

HIV testing and counselling (HTC) represents the first ‘90’ of the *90-90-90* initiative [1] and in South Africa 84.9% of adults living with HIV know their HIV status, however there are concerns that South Africa will not reach this 90% target by 2020 [2]. Established facility-based testing presents barriers to accessing HTC [3–5], and innovative new methods like HIV self-testing (HIVST) are needed close the test gap by reducing barriers for priority populations [6–8]. HIVST refers to a process in which a person performs their own HIV test; which may be more convenient, require less travel and waiting time, and be more private [9–11].

Initially, home-based HIV tests required the user to send a blood sample to a laboratory, then wait days or weeks for results; however modern HIVST rapid diagnostic tests (RDTs) can provide results in minutes, without a laboratory [12]. In 2012 the Food and Drug Administration in the United States approved OraQuick ADVANCE Rapid HIV-1/2 Antibody Test as the first-ever over-the-counter HIVST RDT, including individuals without any prior experience in HIV testing [13].

Growing evidence from different contexts supports the acceptability and usability of HIVST [14–17] and guidelines suggest that only validated products be used for programmes. Since then, 40 countries, including South Africa, have incorporated HIVST in national policies [18] and the South African National Department of Health will only permit the use of WHO pre-qualified products to be used in public health programmes [19]. The WHO prequalification (PQ) process was designed in accordance with international standards, which includes assessing clinical performance among a broad range of self-testing users, with studies that compare the device to a suitable reference standard test [20].

The HIV Self-Testing Assessments and Research (HSTAR) programme, supports HIVST developers in the PQ process, by independently providing data on usability. The purpose of this study was to determine the usability of seven prospective HIVSTs in the hands of untrained, intended users in line with the specifications set out by WHO in the abovementioned prequalification document.

## Methods

### Study design

This usability evaluation was a cross sectional study that used convenience sampling to recruit consenting adults from the general population in the inner-city of Johannesburg, South Africa from June 2016 until June 2018. Using WHO PQ guidance, this study evaluated the usability of seven HIVSTs that were in varying stages of development for the South African market. The HIVST devices were evaluated independently and in series, with one device evaluation finishing before the next started. No participants were enrolled for more than one device.

### HIVSTs

To simulate the real world experience, each HIVST device being evaluated was presented in shelf ready packaging (similar to other devices already approved for distribution within South Africa), using the manufacturer’s instructions for use (IFU) and kit components. Seven HIVST devices were assessed: five fingerstick whole-blood (FS) devices and two oral fluid (OF) devices. The five FS devices were produced by Atomo Diagnostics (Australia) (two devices; generation 1 and generation 2), Biolytical Laboratories (Canada), Biosure Ltd (United Kingdom), and Chembio Diagnostic Systems (USA), while the two OF tests were produced by Calypte Biomedical Corporation (USA) and Orasure Technologies (USA).

### Study participants

Based on the WHO prequalification documents, a sample size of 200 participants [20] was suggested for the usability assessment of each device (1400 total participants). Recruiters were made conscious during training that the study aimed to include diverse age groupings and education levels, as well as equal gender participation [20].

Participants were included if they were 18 years and older, able to read English, first-time HIV self-testers, willing to provide oral fluid or fingerstick blood samples (according to which test was being evaluated) and reported an unknown HIV status. Healthcare workers (including lay counsellors who do HIV testing), any person who had any prior experience with HIV self-testing, persons who had received an experimental HIV vaccine or were taking HIV pre-exposure prophylaxis, and persons known to be HIV positive or have any extenuating condition which may interfere with the process (such as poor vision or intoxication) were excluded. Participants were registered onto a Biometric Enrolment System, which uses fingerprint scanning to eliminate the chance of duplicate enrolment.

### Pilot

At the time of evaluation, no verified or standardized questionnaires for investigating the usability of HIVSTs for prequalification were available, so a product-specific semi-structured questionnaire was developed based on the WHO PQ literature [20–23]. The questionnaire was piloted in a sample of 50 participants for each HIVST device. Findings were shared with the manufacturers, and they were encouraged to incorporate the feedback into their final product for evaluation [16,24]. Not all manufacturers chose to amend their end product, but in some cases, there were major edits to their IFU to ensure clarity and the successful performance of critical steps.

In order to evaluate participants’ actions and perceptions regarding usability, the questionnaire was organized into three parts: a usability index guided by a HIVST process checklist; the interpretation of contrived results; and a post-test interview that assessed the participants’ competency and experiences, while also inviting recommendations (see supporting information S1 Data Collection Checklists).

### Data collection

Participants were handed a “packaged” kit, including the IFU, with no further information about the device or test procedure, then asked to perform the test in front of an observer, who monitored each step of the test for success, confusion, difficulty and errors. The observer remained silent throughout and did not provide any assistance or interference. To document each participants’ actions, an observer used a product-specific semi-structured questionnaire, which included a product-specific checklist and a post-test interview. As this study only evaluated usability, not performance of the HIVST, the HIVST devices were removed after the final processing step (before results could be observed) and substituted for contrived tests that were developed by each manufacturer. Participants were provided with four contrived devices (non-reactive/negative, reactive/positive, weak positive and invalid), serially and in random order, that displayed the possible results and asked to interpret each result.

#### Usability index

Prior studies often describe usability qualitatively, as a way to provide feedback that can be incorporated into future designs, however at the time of this study, there were no validated data collection tools to quantify usability [22,23]. A product-specific HIVST process checklist was developed to calculate a usability index that could be applied to each of the HIVSTs independently. This usability index was motivated by previous HIVST briefing documents from the Blood Products Advisory Committee [25], which quantified operational error rates by identifying and tracking errors based on the IFU. Instead of tracking erroneous steps to identify the error rate (expressed as a percentage), this study tracked successful steps, in order to identify usability with the usability index, which was also expressed as a percentage. The checklist and steps used to calculate each usability index was product-specific, so direct comparisons between HIVSTs could not be made.

In order to calculate the usability index, a device and IFU assessment of each HIVST was used to create a product-specific yes/no checklist of all steps for the HIVST procedure, which ranged from 10 to 17 questions. A trained observer used the checklist to document each participants’ usability of the HIVST by tracking the number of successful participants that completed each step, which was presented as a frequency and percentage. For steps with negative inflection, the ‘No’ response was the value used towards the final usability index (ie. *Was it difficult for the participant to remove the test device from the pouch*?). The successful usability percentages of each step were then averaged, to provide the usability index for each device, from 0% (unusable) to 100% (highly usable) [26,27].

#### Interpreting contrived results

To evaluate the participant’s ability to interpret the device results, contrived tests were provided by each manufacturer to represent the four possible test outcomes: 1) non-reactive/negative, 2) reactive/positive, 3) weak positive, and 4) invalid (no control). Observers used a yes/no checklist to document whether the participant noted the control line and the test line, then whether their interpretation of the results was correct. They rated the participants’ apparent level of confidence and satisfaction, whether the participant appeared calm, nervous, verbally distressed or confused, and whether staff intervention was requested.

HIV test results were not recorded or reported to participants. Participants who wished to have a HIV test were referred to the nearby clinics adjacent to the study sites, where testing is readily available; this is conventional practice in similar studies where HIV results are not made known to participants, for research design reasons, and is a local ethics board requirement.

#### Post-test interview

As part of the post-test interview, participants were asked what to do following both positive and negative results, in order to evaluate how well the participant understood the IFU recommendations for each type of test result.

The post-test interview evaluated each participant’s comprehension of the IFU and packaging information, whether they would use the test again or recommend it to a friend, and experiences throughout the testing process. Open-ended questions asked for comments and recommendations for their test device.

### Data analysis

Data were transcribed from the product-specific semi-structured questionnaire into an excel database by field workers. Quantitative data was analyzed with descriptive statistics in Excel. Qualitative data on usability and experiences were categorized and assessed to provide context and supplement the quantitative results.

### Ethical considerations

The Human Research Ethics Committee of the University of the Witwatersrand provided approval for the study (No. 160306). Once approved, the protocol was registered with the National Human Research Ethics Committee (www.ethicsapp.co.za), the South African National Clinical Trial Registry (www.sanctr.gov.za) and ClinicalTrial.gov. Trained study staff obtained written informed consent from all study participants using an information sheet and informed consent document approved by the Wits HREC. The informed consent form was translated into English, Zulu, Sotho and Xhosa. Participants were given no incentives or reimbursements for their participation in the study.

## Results

While the majority of trial participants were South African (1025/1400(73.0%);58.0%-94.5%), a substantial minority of Zimbabweans and other Africans participated in some of the evaluations. Participant age ranges were evenly distributed with approximately one third being 18–25 years old (493/1400(35%);18.5%-65.0%), 26–35 years old (471/1400(34.0%);15.0%-44.0%) and over 35 years old (435/1400(31.0%);17.5%-48.5%), respectively. Education levels were also distributed relatively evenly, between secondary school (499/1400(36.0%);33.5%-41.0%), tertiary (478/1400(34.0%);33.0%-37.5%) and primary (423/1400(30.0%);25.0%-33.0%). These demographics are summarised in Table 1.

**Table 1:** Participant demographics.

Demographic	Biosure n (%)	INSTI n (%)	Atomo1 n (%)	Atomo2 n (%)	Chembio n (%)	Orasure n (%)	Calypte n (%)	Total n (%)
**Total n**	200 (100.0)	200 (100.0)	200 (100.0)	200 (100.0)	200 (100.0)	200 (100.0)	200 (100.0)	1400 (100.0)
**Nationality**								
South African	129 (64.5)	139 (69.5)	136 (58.0)	189 (94.5)	173 (86.5)	132 (66.0)	127 (63.5)	1025 (73.0)
Zimbabwean	63 (31.5)	55 (27.5)	47 (18.0)	9 (4.5)	21 (10.5)	61 (30.5)	50 (25.0)	306 (22.0)
Other	8 (4.0)	6 (3.0)	17 (19.0)	2 (1.0)	6 (3.0)	7 (3.5)	25 (12.5)	71 (5.0)
**Sex**								
Female	91 (54.5)	104 (52.0)	88 (44.0)	90 (45.0)	80 (40.0)	86 (43.0)	94 (47.0)	633 (44.5)
Male	109 (54.5)	96 (48.0)	112 (56.0)	110 (55.0)	120 (60.0)	114 (57.0)	106 (52.0)	777 (55.5)
**Age**								
18–25 years old	37 (18.5)	54 (27.0)	66 (33.0)	110 (55.0)	130 (65.0)	42 (21.0)	54 (27.0)	493 (35.0)
26–35 years old	88 (44.0)	77 (38.5)	81 (40.5)	55 (27.5)	30 (15.0)	61 (30.5)	80 (40.0)	472 (34.0)
Over 35 years old	75 (37.5)	69 (34.5)	53 (26.5)	35 (17.5)	40 (20.0)	97 (48.5)	66 (33.0)	435 (31.0)
**Education Level**								
Primary school or less	65 (32.5)	66 (33.0)	60 (30.0)	50 (25.0)	51 (25.5)	66 (33.0)	65 (32.5)	423 (30.0)
Secondary school	67 (33.5)	67 (33.5)	74 (37.0)	82 (41.0)	74 (37.0)	67 (33.5)	68 (34.0)	499 (36.0)
Tertiary school (any)	68 (34.0)	67 (33.5)	66 (33.0)	68 (34.0)	75 (37.5)	67 (33.5)	67 (33.5)	478 (34.0)

n-number; %-percentage

### Usability index

The average usability index for all seven HIVSTs was 92.8% (84.2% to 97.6%). Each HIVST has been described individually in further detail below (Table 2) (slightly adapted from the original questionnaire for ease of presenting the results), while the complete datasets for each HIVST are available as supporting information S1–S7 Datasets.

**Table 2:** Usability checklist and index.

Usability Checklists	Yes n (%)	No n (%)	Usability Index (%)
**Biosure**			
1. Did the participant read/use the IFU?	193 (96.5)	7 (3.5)	96.5
2. Did the participant have difficulty removing the test tube from the test pack?	23 (11.5)	177 (88.5)	88.5
3. Did the participant the remove the buffer pot and stand in upright in slot?	148 (74.0)	52 (26.0)	74.0
4. Did the participant have difficulty lancing their finger?	21 (10.5)	179 (89.5)	89.5
5. Did the participant have difficulty forming a blood droplet?	37 (18.5)	163 (81.5)	81.5
6. Was the participant able to fill the tube with adequate amount of blood?	156 (78.0)	44 (22.0)	78.0
7. Was the participant able to push test tube right to the bottom of the buffer pot?	153 (76.5)	47 (23.5)	76.5
8. Was a control line present?	159 (79.5)	41 (20.5)	79.5
9. Did the participant quit the process at any point?	22 (11.0)	178 (89.0)	89.0
10. Did the participant continue the process despite a missed or incorrect step?	178 (89.0)	22 (11)	89.0
Total usability index			**84.2**
**INSTI**			
1. Did the study participant read/use the information sheet?	200 (100.0)	0 (0.0)	100.0
2. Was it difficult for the participant to remove the test device from the pouch?	10 (5.0)	190 (95.0)	95.0
3. Was the study participant able to remove the cap of Bottle 1?	200 (100.0)	0 (0.0)	100.0
4. Did the study participant twist the tip of the lancet off?	200 (100.0)	0 (0.0)	100.0
5. Did the participant rub his/her finger correctly (up and down/vertical motion)?	194 (97.0)	6 (3.0)	97.0
6. Was the study participant able to lance his/her finger correctly?	198 (99.0)	2 (1.0)	99
7. Was the study participant able to form a blood droplet?	188 (94.0)	12 (6.0)	94
8. Was the study participant able to get the blood droplet to fall into Bottle 1?	171 (85.5)	29 (14.5)	85.5
9. Was the study participant able to twist the cap onto Bottle 1?	198 (99.0)	2 (1.0)	99
10. Did the study participant shake Bottle 1, 4 times?	193 (96.5)	7 (3.5)	96.5
11. Did the study participant pour the liquid from Bottle 1 into device and wait until liquid disappeared?	198 (99.0)	2 (1.0)	99
12. Did the study participant shake Bottle 2, 4 times?	193 (96.5)	7 (3.5)	96.5
13. Did the study participant pour the liquid from Bottle 2 into device and wait until liquid disappeared?	198 (99.0)	2 (1.0)	99
14. Did the study participant shake Bottle 3, 4 times?	193 (96.5)	7 (3.5)	96.5
15. Did the study participant pour the liquid from Bottle 3 into device and wait until liquid disappeared?	198 (99.0)	2 (1.0)	99
16. Did the participant quit the process at any point?	1 (0.50)	199 (99.5)	99.5
17. Did the participant continue the process despite a missed or incorrect step?	199 (99.5)	1 (0.5)	99.5
Total usability index			**97.4**
**Atomo1**			
1. Did the study participant read/use the information sheet?	200 (100.0)	0 (0.0)	100.0
2. Was it difficult for the participant to remove the test device from the foil pouch?	2 (1.0)	198 (99.0)	99.0
3. Did the study participant massage finger for 5 to 10 seconds?	200 (100.0)	0 (0.0)	100.0
4. Was the study participant able to gently turn and take out the green tab?	200 (100.0)	0 (0.0)	100.0
5. Did the study participant successfully push the grey button in to prick finger?	195 (97.5)	5 (2.5)	97.5
6. Did the participant place the test device in the section allocated on the IFU?	136 (68.0)	64 (32.0)	68.0
7. Was the study participant able to form a blood droplet by squeezing firmly behind the prick site?	181 (90.5)	19 (9.5)	90.5
8. Did the study participant fill the blood tube with the correct volume of blood?	126 (63.0)	74 (37.0)	63.0
9. Was the participant able to flip the blood tube over into the well, successfully?	181 (90.5)	19 (9.5)	90.5
10. Did the participant ensure the blood has moved from the tube into the well?	126 (63.0)	74 (37.0)	63.0
11. Was the study participant able to pour 3 drops of buffer into the well?	182 (91.0)	18 (9.0)	91.0
12. Did the test fluid run across the strip?	185 (92.5)	15 (7.5)	92.5
13. Did the participant quit the process at any point?	3 (1.5)	197 (98.5)	98.5
14. Did the participant continue the process despite a missed or incorrect step?	187 (93.5)	13 (6.5)	93.5
Total usability index			**89.1**
**Atomo2**			
1. Did the study participant read/use the information sheet?	200 (100.0)	0 (0.0)	100.0
2. Was it difficult for the participant to remove the test device from the foil pouch?	1 (0.5)	199 (99.5)	99.5
3. Did the study participant massage finger for approximately 10 seconds to stimulate the blood flow?	166 (83.0)	34 (17.0)	83.0
4. Was the study participant able to twist the green sterility tab and pull it out?	200 (100.0)	0 (0.0)	100.0
5. Did the participant successfully push hard on the grey button to prick finger?	200 (100.0)	0 (0.0)	100.0
6. Was the study participant able to form a blood droplet by squeezing firmly behind the prick site?	197 (98.5)	3 (1.5)	98.5
7. Did the study participant successfully touch blood to channel?	197 (98.5)	3 (1.5)	98.5
8. Did the study participant adequately fill the channel with enough blood?	186 (93.0)	14 (7.0)	93.0
9. Did the participant successfully push hard on the button to activate the test?	198 (99.0)	2 (1.0)	99.0
10. Did the test fluid run across the strip?	199 (99.5)	1 (0.5)	99.5
11. Did the participant quit the process at any point?	0 (0.0)	200 (100.0)	100.0
12. Did the participant continue the process despite a missed or incorrect step?	200 (100.0)	0 (0.0)	100.0
Total usability index			**97.6**
**Chembio**			
1. Did the study participant read/use the information sheet?	198 (99.0)	2 (1.0)	99.0
2. Was it difficult for the participant to remove the test device from the foil pouch?	6 (3.0)	194 (97.0)	97.0
3. Did the study participant successfully place the stand on a flat surface?	199 (99.5)	1 (0.5)	99.5
4. Was the study participant able to carefully remove the buffer cap?	196 (98.0)	4 (2.0)	98.0
5. Did the participant correctly insert the buffer cap into the test stand?	196 (98.0)	4 (2.0)	98.0
6a. Was the study participant able to open the disinfectant wipe?	194 (97.0)	6 (3.0)	97.0
6b. Was the study participant able to open the sterile pad?	161 (80.5)	39 (19.5)	80.5
7. Did the participant swab finger with disinfectant wipe and allow to dry?	190 (95.0)	10 (5.0)	95.0
8a. Did the study participant successfully uncap safety lancet?	198 (99.0)	2 (1.0)	99.0
8b. Did the study participant successfully place red end of lancet against the side of fingertip?	198 (99.0)	2 (1.0)	99.0
9. Did the participant successfully press down firmly to prick their skin?	197 (98.5)	3 (1.5)	98.5
10. Did the study participant gently squeeze out the first blood drop?	198 (99.0)	2 (1.0)	99.0
11. Did the study participant use the sterile pad to wipe up the blood?	137 (68.5)	63 (31.5)	68.5
12. Did the study participant gently squeeze out second blood drop?	174 (87.0)	26 (13.0)	87.0
13. Was the study participant able to fill the testing device with an adequate amount of blood?	194 (97.0)	6 (3.0)	97.0
14a.Was the study participant able to insert tip of the device into the test stand opening?	194 (97.0)	6 (3.0)	97.0
14b.Was the study participant able to push firmly through the foil cap until 3 snaps were felt?	162 (81.0)	38 (19.0)	81.0
15.Did the participant check for pink stain formation within 1 minute of puncturing the buffer pot?	175 (87.5)	25 (12.5)	87.5
16.Did the participant quit the process at any point?	3 (1.5)	197 (98.5)	98.5
17.Did the participant continue the process despite a missed or incorrect step?	197 (98.5)	3 (1.5)	98.5
Total usability index			**93.7**
**Orasure**			
1. Did the participant read/use the IFU?	200 (100.0)	0 (0.0)	100.0
2. Was it difficult for the study participant to remove the test tube from the pouch?	20 (10.0)	180 (90.0)	90.0
3. Did the study participant remove the cap from the test tube?	196 (98.0)	4 (2.0)	98.0
4. Did the study participant have difficulty with sliding the test tube into the stand?	49 (24.5)	151 (75.5)	75.5
5. Was the study participant able to remove the test device from the pouch?	194 (97.0)	6 (3.0)	97.0
6. Did the study participant touch the flat pad?	27 (13.5)	173 (86.5)	86.5
7. Did the study participant collect the sample correctly?	152 (76.0)	48 (24.0)	76.0
8. Did the study participant place the test device in the test tube correctly?	198 (99.0)	2 (1.0)	99
9. Did the participant quit the process at any point? If Yes, explain	0 (0.0)	200 (100.0)	100
10. Did participant continue the process despite a missed or incorrect step?	200 (100.0)	0 (0.0)	100
Total usability index			**92.2**
**Calypte**			
1. Did the study participant read/use the information sheet?	200 (100.0)	0 (0.0)	100.0
2. Was it difficult for the participant to remove the test contents from the box?	3 (1.5)	197 (98.5)	98.5
3. Did the study participant have difficulty inserting the test tube into the box?	81 (40.5)	119 (59.5)	59.5
4. Did the study participant have difficulty with pulling the cap off the test tube?	24 (12.0)	176 (88.0)	88.0
5. Was the study participant able to remove the oral brush from the plastic bag?	200 (100.0)	0 (0.0)	100.0
6. Did the participant collect the sample correctly (brush 2x upper & 2x lower)?	194 (97.0)	6 (3.0)	97.0
7. Did the study participant insert the oral brush in the test tube correctly?	199 (99.5)	1 (0.5)	99.5
8. Did the study participant slowly push the oral brush up & down, inside the test tube correctly (6–8 X)?	195 (97.5)	5 (2.5)	97.5
9. Did participant squeeze fluid from the oral brush against the test tube correctly?	197 (98.5)	3 (1.5)	98.5
10. Did the study participant remove the oral brush from the test tube?	200 (100.0)	0 (0.0)	100.0
11. Did the study participant remove the test strip from the foil pouch correctly?	198 (99.0)	2 (1.0)	99.0
12. Did the study participant drop the test strip into the test rube correctly (arrows pointing down)?	198 (99.0)	2 (1.0)	99.0
13. Did the participant quit the process at any point?	0 (0.0)	200 (100.0)	100.0
14. Did the participant continue the process despite a missed or incorrect step?	200 (100.0)	0 (0.0)	100.0
Total usability index			**95.5**

n-number; %-percentage; IFU-information for use

#### Biosure

The overall usability index of Biosure was 84.2%. Nearly all participants (193/200;96.5%) read and used the IFU and 23(11.5%) participants experienced difficulties opening the packaging. Twenty one (10.5%) participants had difficulties lancing their finger, while 37(18.5%) participants had difficulties forming a blood droplet, which resulted in only 156(78.0%) participants able to fill the tube with an adequate amount of blood during specimen collection. Approximately one quarter of participants had trouble placing the buffer pot upright in the slot (52/200;26%) and were unable to push the test tube to the bottom of the buffer pot (47/200;23.5%). Despite any missed or incorrect steps, 178(89.0%) participants got to the last step.

#### INSTI

For INSTI, the overall usability index was 97.4%. All (200) participants read and used the information sheet and 10(5.0%) had difficulty removing the device from the packaging. All participants were also able to remove the cap of Bottle 1 and the lancet. With respect to specimen collection, 194(97%) participants correctly massaged their finger, 198(99%) correctly lanced their finger, 188(94%) participants were able to form a bold droplet, and 171(88.5%) were able to successfully transfer the droplet into Bottle 1. Two (1.0%) participants had difficulties capping Bottle 1, seven (3.5%) participants did not shake the bottle the required four times, and only two (1.0%) participants were unable to pour the liquid from Bottle 1 into the device and wait until it disappeared. Similarly, seven (3.5%) participants did not shake the second or third bottle four times, and two (1.0%) participants did not pour the liquid from Bottle 2 or Bottle 3 into the device and wait until it disappeared. Despite any missed or incorrect steps, 199(99.5%) participants completed the entire process.

#### Atomo1

The usability index for Atomo1 was 89.1%. All 200 participants read and used the information sheet and only 2(1.0%) experienced difficulty removing the device from the pouch, however 64(32.0%) were unable to correctly place the device once removed. During specimen collection, all the participants were able to prime the lancet and correctly massage their finger, however 5(2.5%) participants did not successfully prick their finger and 19(8.5%) did not squeeze their finger hard enough to create a blood droplet; this led to only 126(64%) participants filling the blood tube with the correct volume of blood. One hundred and eighty-one (90.5%) participants flipped the blood tube into the well but only 126(63.0%) ensured that the blood successfully moved into the well. The 3 drops of buffer were added correctly by 182(91.0%) participants and the test fluid ran across the strip for 185(92.5%) participants. A total of 187(93.5%) participants completed the entire process, despite any missed or incorrect steps.

#### Atomo2

The Atomo2 usability index was 97.6%. Each of the 200(100%) participants read and used the information sheet and only one (0.5%) had difficulty removing the device from the package. For specimen collection, all participants were able to remove the tab on the lancet and push the grey button to prick the finger. Despite only 163(83.0%) participants having correctly massaged their finger to stimulate blood flow, 197(98.5%) participants were able to form a blood droplet and successfully touch the blood droplet to the channel. Fourteen (7.0%) participants did not produce enough blood to adequately fill the channel, but 198(99.0%) participants were able to press the button to activate the test and the fluid successfully ran across the strip of all but one (0.5%) test. Despite any missed or incorrect steps, all of the participants (200/200;100%) completed the entire process.

#### Chembio

The usability index for Chembio was 93.7%. Almost all participants (198/200;99.0%) read and used the information sheet, while six (3.0%) had difficulty removing the device from the foil package. One hundred and ninety-nine (99.5%) participant successfully set up the stand on a flat surface, while four (2.0%) participants were unable to remove the buffer cap or correctly insert the buffer cap into the test stand. While preparing for specimen collection, six (3.0%) participants did not open the disinfectant wipe, 39(19.5%) had difficulties opening the sterile pad and 10(5.0%) did not disinfect the finger and allow it to dry. While three (1.5%) participants did not push down hard enough to properly prick the skin, almost all participants (198/200;99.0%) successfully uncapped the lancet, correctly placed it against the side of their fingertip and were able to squeeze out the first drop of blood. One hundred and seventy-four (87.0%) were also able to squeeze out a second drop of blood and 194(97.0%) filled the testing device with an adequate amount of blood. After specimen collection, 137(68.5%) participants used the sterile pad to wipe up the blood. Once the specimen was collected, 194(97.0%) participants were able to insert the tip of the device into the test stand opening, 162(81.0%) were able to push firmly through the foil cap (confirmed by 3 snaps) and 175(87.5%) participants checked for the formation of pink stain within one minute of puncturing the buffer pot. A total of 197(98.5%) participants completed the entire process, despite any missed or incorrect steps.

#### Orasure

For Orasure, the overall usability index was 92.2% and all 200 participants read and used the information sheet. Six (3.0%) participants had difficulty removing the test device from the pouch and 20(10.0%) participants had difficulty removing the test tube from the pouch. Once removed, 196(98.0%) were able to remove the test tube cap, and 151(75.5%) were able to slide it into the stand with no difficulty. Despite 27(13.5%) participants inadvertently touching the flat pad, specimen collection was performed correctly by 152(76.0%) participants and 198(99.0%) were able to place the device in the test tube correctly. Despite any missed or incorrect steps, all of the participants (200) completed the entire process.

#### Calypte

The usability index for Calypte was 95.5%. All 200 participants read and used the information sheet and only three (1.5%) experienced difficulty removing the test contents from the box. Eighty-one (40.5%) had difficulty inserting the test tube into the box and 24(12.0%) had difficulty pulling the cap off the test tube. For specimen collection, all participants successfully removed the oral brush from the plastic bag, 194(97.0%) correctly used the brush (brush the upper and lower gums twice each) and 199(99.5%) correctly inserted the brush into the test tube when done collection. One hundred and ninety-five (97.5%) correctly pushed the brush up and down in the test-tube (six to eight times), 197(98.5%) correctly squeezed fluid from the brush correctly, then all of the participants correctly removed the brush from the test tube. Once the brush was removed, 198(99.0%) participants properly removed the test strip from the foil pouch and correctly dropped it into the test tube. All 200(100%) participants completed the entire process, despite any missed or incorrect steps.

#### Interpreting contrived results

Overall, participants could correctly interpret the non-reactive/negative (1346/1400(96.1%);91.0%-99.5%) and reactive/positive results (1357/1400(97.0%);94.5%-99.5%) accurately for each of the devices (Table 3). Weak reactive/positive results had a much lower number of total correct interpretations (1048/1400(74.9%);49.5%-94.0%) and most commonly, weak positive results were interpreted as non-reactive/negative. The invalid test result was read correctly in most cases (1290/1400(92.1%);83.0%-98.0%).

**Table 3:** Contrived results, post-testing and user perceptions.

Outcomes	Biosure	INSTI (%)	Atomo1	Atomo2	Chembio	Orasure	Calypte	Total
Interpreting contrived results	n (%)	n (%)	n (%)	n (%)	n (%)	n (%)	n (%)	n (%)
Non-reactive / Negative	186 (93.0)	198 (99)	199 (99.5)	195 (97.5)	188 (94.0)	182 (91.0)	198 (99.0)	1346 (96.1)
Reactive / Positive	189 (94.5)	199 (99.5)	198 (99.0)	191 (95.5)	191 (95.5)	192 (96)	197 (98.5)	1357 (97.0)
Weak reactive / Weak positive	149 (74.5)	188 (94.0)	173 (86.5)	173 (86.5)	126 (63.0)	99 (49.5)	140 (70.0)	1048 (74.9)
Invalid (no control line)	173 (86.5)	194 (97.0)	191 (95.5)	184 (92.0)	166 (83.0)	186 (93.0)	196 (98.0)	1290 (92.1)
**What to do after HIVST**								
***If Negative/Non-Reactive***								
Re-test in 3 months	59 (29.5)	162 (81.0)	162 (81.0)	60 (30.0)	1 (0.5)	100 (50.0)	165 (82.5)	709 (50.6)
Condomize	87 (43.5)	26 (13.0)	32 (16.0)	134 (67.0)	138 (69)	54 (27.0)	35 (17.5)	506 (36.1)
Other	54 (27.0)	12 (6.0)	6 (3.0)	6 (3.0)	61 (30.5)	46 (23.0)	0 (0.0)	185 (13.2)
***If Positive/Reactive***								
Visit clinic/seek treatment/counselling	189 (94.5)	198 (99.0)	199 (99.5)	195 (97.5)	193 (96.5)	184 (92.0)	200 (100.0)	1358 (97.0)
Other	11 (5.5)	2 (1.0)	1 (0.5)	5 (2.5)	7 (3.5)	16 (8.0)	0 (0.0)	42 (3.0)
**Participant perceptions of HIVST process**								
Were the Instructions for Use easy to understand?	186 (93.0)	198 (99.0)	197 (98.5)	199 (99.5)	196 (98.0)	198 (99.0)	196 (98.0)	1370 (97.9)
Was the device easy to use?	184 (92.0)	197 (98.5)	185 (92.5)	200 (100.0)	197 (98.5)	192 (96.0)	197 (98.5)	1352 (96.6)
Are you confident with performing this test on your own?	172 (86.0)	193 (96.5)	197 (98.5)	194 (97.0)	192 (96.0)	197 (98.5)	197 (98.5)	1342 (95.9)
Would you prefer to use this test at home? ((vs. get tested at a clinic)	162 (81.0)	160 (80.0)	162 (81.0)	166 (83.0)	159 (79.5)	167 (83.5)	155 (77.5)	1131 (80.1)
Will you use this test again?	193 (96.5)	172 (86.0)	194 (97.0)	192 (96.0)	193 (96.5)	200 (100.0)	200 (100.0)	1344 (96.0)
Will you recommend this test to a sexual partner/friend?	194 (97.0)	195 (97.5)	195 (97.5)	191 (95.5)	195 (97.5)	196 (98.0)	195 (97.5)	1361 (97.2)

n-number; %-percentage

#### Post HIVST

When asked what to do after testing positive, many of the participants (1358/1400(97.0%);92.0%-100.0%) stated that they would go to a clinic or seek treatment. For negative results, approximately half the participants (709/1400 (50.6%);0.5%-82.5%) stated that they should re-test in the next three months, while just over one third (506/1400 (36.1%);13.0%-69.0%) reported that they should condomize (Table 3).

#### Participant perceptions of the HIVST process

Nearly all of the participants found the device easy to use (1352/1400 (96.6%);92.0%-100.0%) and found the IFUs to be easy to understand (1370/1400(97.9%);93.0%-99.5%) (Table 3). Many participants (1342/1400(95.9%);86.0%-98.5%) felt confident using the device on their own and would prefer using the test at home instead of a clinic (1131/1400 (80.1%);77.5%-83.5%). Almost all participants (1344/1400 (96.0%);86.0%-100.0%) would use the test again and (1361/1400 (97.2%);95.5%-95.5%) would recommend the test to a partner or friend.

Participants acknowledged difficulty with some steps due to kit engineering (e.g. difficulty assembling the device, or fitting parts together), particularly where there was a tight fit or firm pressure was required. Participants made suggestions where some issues could be resolved by the IFU–asking for some steps to be clarified or emphasized in places:

“*Some steps were hard to follow*, *pictures help”*

- Male, 25 years old, FS

When asked what they liked least about their HIV self-test, participants in the FS studies identified a general dislike of the needle, however this was reported by less than 12% (6%-11%) of the fingerstick participants. Many participants had never used a safety lancet and were nervous about the needle, but then expressed surprise that it wasn’t as painful as anticipated. Some participants expressed frustration with the fingerstick process (“*needle not working”* and “*needle not sharp enough”*) and could not acquire enough sample (particularly noted for device Atomo1). For the fingerstick step, participants suggested improved instruction to successfully apply the lancet (“*press firmly”* with a picture of the correct location) and to obtain the necessary sample volume (“*keep squeezing”* to form blood droplet):

“Safety lancet is bit confusing; the producer should make it more visible.”

- Male, 48 years old, FS

For the OF studies, participants had minimal apprehension about obtaining a specimen; most of the complaints focused on difficulties with assembling the kit components (*“cap is too tight”* and *“where to insert the tube”*). When asked what they liked best about their HIV self-test, both OF and FS participants overwhelmingly liked the convenience and confidentiality of a self-test. Approximately 7% of OF self-testers specifically stated that that they like that their test required “*no needle”* and “*no blood”* (though four OF participants stated a higher confidence for blood-based testing).

Both OF and FS participants preferred home-based self-testing to clinic-based testing, and appreciated that the HIV self-test was confidential, fast, did not require clinic queues and gave them autonomy for their health decisions.

“Home test is easier and less scary compared to clinic.”

- Male, 30 years old, OF

Several participants expressed a desire for a trained professional to be available to assist in using the device (both FS and OF), if necessary, while others were concerned about the lack of counselling available with the HIVST.

*“Before the client buy this product*, *the must be someone to demonstrate to the clients at pharmacy on how to use this product.”*

- Male, 19 years old, OF

“Where there is no counselling wrong decisions can be made.”

- Male, 30 years old, FS

## Discussion

While this is the first large-scale usability study incorporating multiple HIVSTS in South Africa, the strong usability outcomes, are consistent with recent studies conducted in other populations. A study of the Exacto Test HIV in the Central African Republic showed that 91% of participants found the test easy to use and 91.6% performed the test correctly, however 23% asked for oral assistance [28]. Similarly, a Kenyan INSTI usability study showed that 94.3% of participants found the test easy to use [23]. The high average usability index of 93.8% is further corroborated by 96.6% of all participants stating that the HIVSTs were easy to use.

Despite the high usability scores, this evaluation also provided observations and perspectives that may improve upon current offerings by identifying points of confusion or hesitation, as well as any critical and non-critical errors made during the self-testing process. For each device, difficulties with packaging, instructions, and/or kit components were identified for improvement to increase ease-of-use and reduce misuse of the self-test. Each device manufacturer received a final usability report to help assess product readiness for the HIV self-testing market in South Africa, along with recommendations for any HIVST kit improvements.

User errors were high when participants had difficulty obtaining and transferring the specimen. For the FS devices, the most common specimen collection errors included lancing mistakes, not acquiring a sufficient blood droplet, or not adequately filling the transfer capillary. These results were comparable to a previous usability study of Atomo1 in adolescents from Cape Town, South Africa [21]. Errors in FS sample acquisition might be decreased through improvements to the IFU and/or more reliable lancets. For the OF devices, the most common specimen collection errors came from incorrectly swabbing the mouth, which could also be decreased through improvements to the IFU. Since no diagnostic results were collected, these user errors cannot be directly linked to incomplete or incorrect test results; follow-on studies of full diagnostic performance are currently underway.

As participant responses suggest, it would be beneficial to have a choice between whole blood or oral fluid HIVST device, as different users have indicated a preference for both; one kind of self-test modality is unlikely to suit everyone. This variance was also observed in a recent study of men who have sex with men (MSM) in Mpumalanga, South Africa, which showed that OF tests (OraSure) were easier to use, while the FS tests (Atomo) were preferred by participants [29]. Even with the choice of OF and FS devices, self-testing alone, is unlikely to be beneficial for everyone who needs an HIV screening test, as roughly 20% of all participants indicated they were uncomfortable performing the test without guidance or counselling at hand. This proportion was similar across 7 devices, suggesting that people’s views on this are unrelated to any technical aspect of HIVST devices and may instead indicate a social preference. Similar findings were published in an OraQuik study from Singapore, which suggested that the acceptability of HIVSTs may be influenced by socioeconomic status [30]. Age may also play a factor, as the Cape Town adolescent usability study showed slightly lower usability scores with a similar style data collection tool, as participants scored the device 4 out of a maximum rating of five [21]. A multi-dimensional approach including home-testing, community-supported testing and clinic-based testing will likely be needed to reach key and under-tested populations, including men, adolescents and serodiscordant partners. The ability to privately and easily perform an HIV self-test is a much-needed innovation to enable earlier diagnosis of HIV and empower individuals to monitor their own health and behaviour.

Although not part of the usability index, the interpretation of contrived results and what to do after testing also identified some areas of possible improvement. While most participants correctly interpreted non-reactive/negative, reactive/positive and invalid (no control line) results, one quarter of all participants misinterpreted the weak reactive/positive, most commonly as non-reactive/negative. The results for the positive, negative and invalid tests were similar to the Central African Public study, however that study did not investigate the interpretation of weak positive results [28]. The contrived weak positive results ranged in intensity and were specific to each product, so some device weak results may have appeared feint or lighter than others. Most of the IFUs provided simple recommendations for test results with the pictured examples, such as “go to clinic” for a reactive/positive result, and “re-test in 3 months” for a non-reactive/negative result, however some IFUs did not include recommendations for the non-reactive/negative or invalid test results. We noted that participants achieved the most accurate result interpretation when the test device could be placed next to “life sized” examples of the possible test outcomes in the IFU. In order to achieve the most accurate result interpretation, “life sized” comparison examples of all possible test outcomes should be prominently displayed in the IFUs of each HIVST, a suggestion that was also made by Bwana et al in the Kenyan study, as that IFU also lacked a weak positive image [23]. This should also be coupled with simple IFU recommendations of what to do after testing, in the case of each possible result.

The results of the HSTAR001 Usability study presents general guidelines for safe and easy-to-use HIVST test kits including minimizing packaging and clearly marking kit components, simplifying and ordering IFU steps (placing particular emphasis on key steps where necessary), minimizing the number of words used in favour of simple pictures and universal icons (e.g. for the FS have a picture of the target (finger) and orientation of lancet, emphasizing the need to press firmly and for oral fluid to include a picture how the user must place or move tool inside their mouth), providing interpretation of all test outcomes and including “life size” pictures for device comparison, and clearly indicating the next steps for each type of test outcome.

### Limitations

This study presented some limitations. The convenience sampling may present a selection bias, as there were different proportions of sub-demographics between devices, which may have been due to the different communities surrounding each study location. The evaluation of the devices in series ensured no cross-contamination, however the participants from the community may have become more aware of HIVST by the time the last device was tested. For data collection, there is no validated or standardized usability test for HIVSTs, so the product-specific semi-structured questionnaire was developed internally and used to quantify usability. This questionnaire only allowed for each device to be evaluated independently. No direct comparisons between products could be made, as a result of different device components and IFUs not being standardized across kits. For example, there was no universal standard for intensity of a weak positive used to test readings of contrived results. The WHO prequalification only requires independent data on usability and does not require any direct comparisons between products, however we are working on developing a more standardized tool as a separate project as comparisons would be beneficial as more HIV self-tests reach the market.

The usability and comprehension of test instructions are also likely to be context and population specific (for instance, high levels of participants preferring English IFUs), limiting the generalisability of these findings. Some responses may have been influenced by participants’ prior HIV testing experience and post-test counselling prior to this study, which is high in the sampling area. Since no diagnostic results were collected, we could not ascertain whether the user errors identified in the study led to incomplete or incorrect test results in this usability study; follow-on studies of full diagnostic performance are currently underway.

## Conclusions

The results of the HSTAR001 HIVST Usability studies indicate that several devices show overall high levels of usability (ease-of-use) and acceptability in the hands of lay people. Correlating these usability results to test outcome and diagnostic accuracy is the next step in forthcoming studies.

Each of these 7 HIVST devices evaluated in this study is intended to enter the South African HIVST market–and more are likely to follow. Several HIVST candidates are close to final evaluation of test performance and international regulatory approval, and one has recently achieved approval from the WHO PQ (Orasure). Lessons learnt from this evaluation have resulted in guidelines for use, which if taken up by manufacturers will assist in their HIVST candidates achieving WHO PQ Test design, usability and performance, however, are only the first step; to pave the way for HIVST uptake, there needs to be policy engagement to approve and support distribution channels appropriate to both the private pharmacy-based and public health markets.

## Supporting information

S1 Data Collection Checklists (PDF)

S1 Dataset Atomo Gen1 Dataset. (XLSX)

S2 Dataset Atomo Gen2 Dataset. (XLSX)

S3 Dataset Biosure Dataset. (XLSX)

S4 Dataset Calypte Dataset. (XLSX)

S5 Dataset Chembio Dataset. (XLSX)

S6 Dataset INSTI Dataset. (XLSX)

S7 Dataset Orasure Dataset. (XLSX)
