# Supplementary material for: Usability assessment of seven HIV self-test devices conducted with lay-users in Johannesburg, South Africa
Source: PLoS One. 2020 Jan 14;15(1):e0227198. doi: 10.1371/journal.pone.0227198 (PMC6959591; doi:10.1371/journal.pone.0227198)
Supplement: S1 Data Collection Checklists — (PDF) [file pone.0227198.s001.pdf]

HSTAR001 Data Collection Form

**Device type: Atomo**			**Researcher:**		**Date:**	
**Participant code:** ___ ___ ___ - ___ ___ ___ ___ - ___						
**Age:**	18-25 ☐	26-35 ☐	36-45 ☐	46-55 ☐	56-65 ☐	>65 ☐
**Dominant hand:**	Right ☐	Left ☐	**Gender:**	Male ☐	Female ☐	
**Education level:**	☐ ≤Grade 7 primary schooling level		☐ ≥Grade 8 primary schooling to ≤matric level		☐ ≥Technikon, University and University plus	

Section A. Test Performance

Process START time: ____ ____ : ____ ____

1. Did the study participant read/use the information sheet?
   Yes ☐
   No ☐

   If yes, was the IFU read before the test?

   Yes ☐
   No ☐

   Was it referred to during the test process?

   Yes ☐
   No ☐

   ____
   ____
   ____

2. Was it difficult for the study participant to remove the test device from the foil pouch?
   Yes ☐
   No ☐
   If Yes then, what seemed to be the problem? (E.g. could not find tear point, weakness, confusion)

   ____

3. Did the study participant massage finger for approximately 10 seconds to stimulate blood flow?

Yes ☐

No ☐

If No, describe what was done?

___

4. Was the study participant able twist the green sterility tab and pull it out?

Yes ☐

No ☐

If No, describe what was done?

___

5. Did the study participant successfully push hard on the grey button to prick finger?

Yes ☐

No ☐

If No, describe what seemed to be the problem?

___

6. Was the study participant able to form a blood droplet by squeezing firmly behind the prick site?

Yes ☐

No ☐

If No, describe what was done?

___

7. Did the study participant successfully touch blood to channel?

Yes ☐
No ☐
If No then, describe what seemed to be the problem?

____
____
____

8. Did the study participant adequately fill the channel with enough blood?
Yes ☐
No ☐
If No then, describe what was done?

____
____
____

9. Did the study participant successfully push hard on the button to activate the test?
Yes ☐
No ☐
If No then, describe what was done?

____
____
____

10. Did the test fluid run across the strip?
Yes ☐
No ☐
If No then, describe what was done?

____
____
____

Process END time: ____ ____ : ____ ____

11. Did the participant quit the process at any point? If Yes, explain

____

____

12. Did the participant continue the process despite a missed or incorrect step?

____

____

Any other observer comments:
NOTES: (Was there significant hesitation or indecision at specific steps or overall? Did they say anything; did they ask any questions of the interviewer during the process? Which steps were skipped or modified?)

____

____

____

____

Section B. Result Interpretation

1. What did the participant interpret the following results as?

Negative / Non-reactive

Negative ☐
Positive ☐
Invalid/test did not work ☐
Do not know/Not sure ☐
Other____

Faint positive / Low-reactive

Negative ☐
Positive ☐
Invalid/test did not work ☐
Do not know/Not sure ☐
Other____

Positive / Reactive

Negative ☐
Positive ☐
Invalid/test did not work ☐
Do not know/Not sure ☐
Other____

Invalid (NO lines)

Negative ☐
Positive ☐
Invalid/test did not work ☐
Do not know/Not sure ☐
Other____

Invalid (Test line ONLY)

Negative ☐
Positive ☐
Invalid/test did not work ☐
Do not know/Not sure ☐
Other____

Additional Interpretation Comments:

____
____
____

Section C. Post-test Interview

1. When was the last time you had an HIV test done?
   ____
2. Did you use the Instructions sheet? ____
   If NO, please explain

____
____
____

3. Were the instructions easy to follow? Were the pictures helpful?

____
____
____

4. Was the device easy to use? ____
   If NO, please explain the steps that were difficult or confusing

____
____
____

5. How long should you wait before reading your result? ___________________________

6. Were you confident with performing this test on your own? ____________________

If NO, please explain why you were not?

___

___

___

7. Was it easy for you to INTERPRET the mock results? If NO, please explain (*Interviewer indicate which mock device they are referring to*)

___

___

___

8. What should you do if you have a negative result?

___

___

___

9. What should you do if you have a positive result?

___

___

___

10. What should you do if your test did not work?

___

___

___

11. How likely are you to use this test again?
(Please tick one of the boxes below)

I will not use it	Unlikely	Unsure	Likely	Definitely use it

12. Would you prefer to use this test at home or get tested at a clinic?
(Please tick one of the boxes below)

Home	Clinic	Neither

13. How likely are you to recommend this test to a sexual partner, friend, or family member?
(Please tick one of the boxes below)

I will not recommend it	Unlikely	Unsure	Likely	Definitely recommend it

14. What are your favourite things about this test?

____
____
____

15. What are your least favourite things about this test?

____
____
____

HIV Self Testing Assessments & Research

HSTAR001 Data Collection Form

**Device type: Atomo**			**Researcher:**		**Date:**	
**Participant code:** ___ ___ ___ - ___ ___ ___ ___ - ___						
**Age:**	18-25 ☐	26-35 ☐	36-45 ☐	46-55 ☐	56-65 ☐	>65 ☐
**Dominant hand:**	Right ☐	Left ☐	**Gender:**	Male ☐	Female ☐	
**Education level:**	☐ ≤Grade 7 primary schooling level		☐ ≥Grade 8 primary schooling to ≤matric level		☐ ≥Technikon, University and University plus	

Section A. Test Performance

Process START time: ____ ____ : ____ ____

1. Did the study participant read/use the information sheet?
   Yes ☐
   No ☐

   If yes, was the IFU read before the test?

   Yes ☐
   No ☐

   Was it referred to during the test process?

   Yes ☐
   No ☐

   ____
   ____
   ____

2. Was it difficult for the study participant to remove the test device from the foil pouch?
   Yes ☐
   No ☐
   If Yes then, what seemed to be the problem? (E.g. could not find tear point, weakness, confusion)

   ____
   ____
   ____

3. Did the study participant massage finger for 5 to 10 seconds?

Yes ☐

No ☐

If No, describe what was done?

____

____

____

4. Was the study participant able to gently turn and take out the green tab?

Yes ☐

No ☐

If No, describe what seemed to be the problem?

____

____

____

5. Did the study participant successfully push the grey button in to prick finger?

Yes ☐

No ☐

If No, describe what seemed to be the problem?

____

____

____

6. Did the study participant place the test device in the section allocated on the IFU?

Yes ☐

No ☐

If No, describe what was done?

____

____

____

7. Was the study participant able to form a blood droplet by squeezing firmly behind the prick site?

Yes ☐

No ☐

If No then, describe what was done?

____

____

____

8. Did the study participant fill the blood tube with the correct volume of blood?
   Yes ☐
   No ☐
   If No then, describe what seemed to be the problem?
   ____
   ____
   ____

9. Was the study participant able to flip the blood tube over into the well, successfully?
   Yes ☐
   No ☐
   If No then, describe what was done?
   ____
   ____
   ____

10. Did the study participant ensure the blood has moved from the tube into the well?
    Yes ☐
    No ☐
    If No then, describe what was done?
    ____
    ____
    ____

11. Was the study participant able to pour 3 drops of buffer into the well?
    Yes ☐
    No ☐
    If No then, describe what was done?
    ____
    ____
    ____

12. Did the test fluid run across the strip?
    Yes ☐
    No ☐
    If No then, describe what was done?

____
____
____

Process END time: ____ ____ : ____ ____

13. Did the participant quit the process at any point? If Yes, explain
____
____

14. Did the participant continue the process despite a missed or incorrect step?
____
____

Any other observer comments:
NOTES: (Was there significant hesitation or indecision at specific steps or overall? Did they say anything; did they ask any questions of the interviewer during the process? Which steps were skipped or modified?)
____
____
____
____

Section B. Result Interpretation

1. What did the participant interpret the following results as?

Negative / Non-reactive

Negative ☐
Positive ☐
Invalid/test did not work ☐
Do not know/Not sure ☐
Other____

Faint positive / Low-reactive

Negative ☐
Positive ☐
Invalid/test did not work ☐
Do not know/Not sure ☐
Other____

Positive / Reactive

Negative ☐
Positive ☐
Invalid/test did not work ☐
Do not know/Not sure ☐
Other____

Invalid

Negative ☐
Positive ☐
Invalid/test did not work ☐
Do not know/Not sure ☐
Other____

Additional Interpretation Comments:

____
____
____

Section C. Post-test Interview

HIV Self Testing Assessments & Research

1. When was the last time you had an HIV test done?
   ____
2. Did you use the Instructions sheet? ______________________________
   If NO, please explain

____
____
____

3. Were the instructions easy to follow? Were the pictures helpful?

____
____
____

4. Was the device easy to use? ______________________________
   If NO, please explain the steps that were difficult or confusing

____
____
____

5. How long should you wait before reading your result? ____________________

6. Were you confident with performing this test on your own? ____________
   If NO, please explain why you were not?

____
____
____

7. Was it easy for you to INTERPRET the mock results? If NO, please explain (*Interviewer indicate which mock device they are referring to*)

____
____
____

8. What should you do if you have a negative result?

____
____
____

9. What should you do if you have a positive result?

____
____
____

10. What should you do if your test did not work?

____

____

____

11. How likely are you to use this test again?
(Please tick one of the boxes below)

I will not use it	Unlikely	Unsure	Likely	Definitely use it

12. Would you prefer to use this test at home or get tested at a clinic?
(Please tick one of the boxes below)

Home	Clinic	Neither

13. How likely are you to recommend this test to a sexual partner, friend, or family member?
(Please tick one of the boxes below)

I will not recommend it	Unlikely	Unsure	Likely	Definitely recommend it

14. What are your favourite things about this test?

____

____

____

15. What are your least favourite things about this test?

____

____

____

HSTAR
HIV Self Testing Assessments & Research

HSTAR001 Data Collection Form

**Device type: Biosure**			**Researcher:**		**Date:**	
**Participant code:** ___ ___ ___ - ___ ___ ___ ___ - ___						
**Age:**	18-25 ☐	26-35 ☐	36-45 ☐	46-55 ☐	56-65 ☐	>65 ☐
**Dominant hand:**	Right ☐	Left ☐	**Gender:**	Male ☐	Female ☐	
**Education level:**	☐ ≤Grade 7 primary schooling level		☐ ≥Grade 8 primary schooling to ≤matric level		☐ ≥Technikon, University and University plus	

Section A. Test Performance

Process START time: _____ _____ : _____ _____

1. Did the study participant read/use the information sheet?
   Yes ☐
   No ☐

   If yes, was the IFU read before the test?

   Yes ☐
   No ☐

   Was it referred to during the test process?

   Yes ☐
   No ☐

   ____
   ____
   ____

2. Was it difficult for the study participant to remove the test tube from the pouch?
   Yes ☐
   No ☐
   If Yes then, what seemed to be the problem? (E.g. could not find tear point, weakness, confusion)

   ____

HIV Self Testing Assessments & Research

____

____

3. Was the study participant able to remove the buffer pot and stand it upright in the hole provided?

Yes ☐
No ☐
If No then how was it done?

____
____
____

4. Did the study participant have difficulty with lancing his/her finger?

Yes ☐
No ☐
If Yes describe what seemed to be the problem?

____
____
____

5. Did the study participant have difficulty forming a blood droplet?

Yes ☐
No ☐
If Yes describe what seemed to be the problem?

____
____
____

6. Was the study participant able to fill the capillary with an adequate amount of blood?

Yes ☐
No ☐
If No describe what seemed to be the problem?

____
____
____

7. Was the study participant able to push the test tube right to the bottom of the buffer pot?

Yes ☐
No ☐
If No then, how was it done?

____

____
____

8. Did the control line appear?
   Yes ☐
   No ☐

   Any other observer comments
   ____
   ____
   ____

Process END time: ____ ____ : ____ ____

9. Did the participant quit the process at any point? If Yes, explain
   ____
   ____

10. Did the participant continue the process despite a missed or incorrect step?
   ____
   ____

   Any other observer comments:
   NOTES: (Was there significant hesitation or indecision at specific steps or overall? Did they say anything; did they ask any questions of the interviewer during the process? Which steps were skipped or modified?)
   ____
   ____
   ____
   ____

Section B. Result Interpretation

1. What did the participant interpret the following results as?

Negative / Non-reactive

Negative ☐
Positive ☐
Invalid/test did not work ☐
Do not know/Not sure ☐
Other____

Faint positive / Low-reactive

Negative ☐
Positive ☐
Invalid/test did not work ☐
Do not know/Not sure ☐
Other____

Positive / Reactive

Negative ☐
Positive ☐
Invalid/test did not work ☐
Do not know/Not sure ☐
Other____

Invalid

Negative ☐
Positive ☐
Invalid/test did not work ☐
Do not know/Not sure ☐
Other____

Additional Interpretation Comments:

____

___

___

___

___

___

Section C. Post-test Interview

1. When was the last time you had an HIV test done?
   ___
2. Did you use the Instructions sheet? ___
   If NO, please explain

___

___

___

3. Were the instructions easy to follow? Were the pictures helpful?

___

___

___

4. Was the device easy to use? ___
   If NO, please explain the steps that were difficult or confusing

___

___

___

5. How long should you wait before reading your result?

6. Were you confident with performing this test on your own? ___
   If NO, please explain why you were not?

___

___

___

7. Was it easy for you to INTERPRET the mock results? If NO, please explain (*Interviewer indicate which mock device they are referring to*)

___

___

___

8. What should you do if you have a negative result?

9. What should you do if you have a positive result?

10. Would you use this test again?

11. Would you prefer to use this test at home or get tested at a clinic?

12. Would you recommend this test to a sexual partner/friend?

13. What are your 3 favourite things about this test?

14. What are your least favourite things about this test?

HSTAR001 Chembio Data Collection Form

PLACE YOUR BARCODE HERE

**Device type: Chembio**			**Researcher:**		**Date:**	
**Participant code:** ___ ___ ___ - ___ ___ ___ ___ - ___						
**Age:**	18-25 ☐	26-35 ☐	36-45 ☐	46-55 ☐	56-65 ☐	>65 ☐
**Dominant hand:**	Right ☐	Left ☐	**Gender:**	Male ☐	Female ☐	
**Education level:**	☐ ≤Grade 7 primary schooling level		☐ ≥Grade 8 primary schooling to ≤matric level		☐ ≥Technikon, University and University plus	

Section A. Test Performance

Process START time: ____ ____ : ____ ____

1. Did the study participant read/use the information sheet?

   Yes ☐
   No ☐

   If yes, was the IFU read before the test?

   Yes ☐
   No ☐

   Was it referred to during the test process?

   Yes ☐
   No ☐

   ____
   ____
   ____

2. Was it difficult for the study participant to remove the test device from the foil pouch?

   Yes ☐
   No ☐

HSTAR
HIV Self Testing Assessments & Research

RHI

HSTAR001 Data Collection Form

**Device type: CALYPTE**			**Researcher:**		**Date:**	
**Participant code:** ___ ___ ___ - ___ ___ ___ ___ - ___						
**Age:**	18-25 ☐	26-35 ☐	36-45 ☐	46-55 ☐	56-65 ☐	>65 ☐
**Dominant hand:**	Right ☐	Left ☐	**Gender:**	Male ☐	Female ☐	
**Education level:**	☐ ≤Grade 7 primary schooling level		☐ ≥Grade 8 primary schooling to ≤matric level		☐ ≥Technikon, University and University plus	

Section A. Test Performance

Process START time: _____ _____ : _____ _____

1. Did the study participant read/use the information sheet?
   Yes ☐
   No ☐

   If yes, was the IFU read before the test?

   Yes ☐
   No ☐

   Was it referred to during the test process?

   Yes ☐
   No ☐

   ____
   ____
   ____

2. Was it difficult for the study participant to remove the test contents from the box?
   Yes ☐
   No ☐
   If Yes then, what seemed to be the problem? (e.g. couldn't find opening, weakness, confusion)

   ____

____
____

3. Did the study participant have difficulty inserting test tube into the test box?
   Yes ☐
   No ☐
   If Yes then, why?
   ____
   ____
   ____

4. Did the study participant have difficulty with pulling the cap off the test tube?
   Yes ☐
   No ☐
   If Yes then, why?
   ____
   ____
   ____

5. Was the study participant able to remove the oral brush from the plastic bag?
   Yes ☐
   No ☐
   If No then, what seemed to be the problem?
   ____
   ____
   ____

6. Did the study participant collect the sample correctly (brush 2x upper and 2x lower)?
   Yes ☐
   No ☐
   If No then, what did they do?
   ____
   ____
   ____

7. Did the study participant insert the oral brush in the test tube correctly?
   Yes ☐
   No ☐
   If No then, how was it done?
   ____
   ____
   ____

8. Did the study participant slowly push the oral brush up and down, inside the test tube correctly (6-8 X)?
   Yes ☐
   No ☐
   If No then, how was it done?
   ____
   ____
   ____

9. Did the study participant squeeze fluid from the oral brush against the test tube correctly?
   Yes ☐
   No ☐
   If No then, how was it done?
   ____
   ____
   ____

10. Did the study participant remove the oral brush from the test tube?
   Yes ☐
   No ☐
   If No then, what did they do?
   ____
   ____
   ____

11. Did the study participant remove the test strip from the foil pouch correctly?
   Yes ☐
   No ☐
   If No then, what did they do?
   ____
   ____
   ____

12. Did the study participant drop the test strip into the test tube correctly (arrows pointing down)?
   Yes ☐
   No ☐
   If No then, how was it done?
   ____
   ____
   ____

13. Should you remove the test strip before reading the results? ________________________
    Yes ☐
    No ☐

14. Does the IFU show you where the test strip lines / bands should be?
    Yes ☐
    No ☐

15. Did the control line appear? ____
    Yes ☐
    No ☐

Process END time: ____ ____ : ____ ____

16. Which language was preferred by the user? _____________________________________

17. Did the participant quit the process at any point? If Yes, explain
    ____
    ____

18. Did the participant continue the process despite a missed or incorrect step?
    ____
    ____

Any other observer comments:
NOTES: (Was there significant hesitation or indecision at specific steps or overall? Did they say anything; did they ask any questions of the interviewer during the process? Which steps were skipped or modified?)
____
____
____
____

Section B. Result Interpretation

1. What did the participant interpret the following results as?

Negative / Non-reactive

Negative ☐
Positive ☐
Invalid/test did not work ☐
Do not know/Not sure ☐
Other____

Faint positive / Low-reactive

Negative ☐
Positive ☐
Invalid/test did not work ☐
Do not know/Not sure ☐
Other____

Positive / Reactive

Negative ☐
Positive ☐
Invalid/test did not work ☐
Do not know/Not sure ☐
Other____

Invalid

Negative ☐
Positive ☐
Invalid/test did not work ☐
Do not know/Not sure ☐
Other____

Additional Interpretation Comments:

____
____
____

Section C. Post-test Interview

1. When was the last time you had an HIV test ?
   ____

2. Can you locate the storage instructions on the box?
   ____

3. Should you use the test kit after the expiry date?
   ____

4. Did you use the Instructions sheet?
   If NO, please explain

____
____
____

5. Were the instructions easy to follow? Were the pictures helpful?

____
____
____

6. Was the labelling easy to follow?
   ____

7. Was the device easy to use?
   ____
   If NO, please explain the steps that were difficult or confusing

____
____
____

8. Should you use this test if you had anything to eat or drink recently?
   ____

9. How long should you wait before reading the test?
   ____

10. What is the maximum time to read the test result?
    ____

11. How should you dispose of a used test kit?
    ____

12. Are you confident with performing this test on your own? ______________________
    If NO, please explain why you were not?

____
____
____

13. Was it easy for you to INTERPRET the mock results? If NO, please explain (*Interviewer indicate which mock device they are referring to*)

____
____
____

14. What should you do if you have a negative result?

____
____
____

15. What should you do if you have a positive result?

____
____
____

16. What should you do if your test did not work result?

____
____
____

17. What should you do if you are not sure of your result?

____
____
____

18. Would you use this test again?

____

19. Would you prefer to use this test at home or get tested at a clinic?

____

20. Would you recommend this test to a sexual partner/friend?

____

21. What are your 3 favourite things about this test?

____
____
____

22. What are your least favourite things about this test?

____
____
____

If Yes, what seemed to be the problem? (E.g. could not find tear point, weakness, confusion)

____

____

____

3. Did the study participant successfully place stand on flat surface?

Yes ☐

No ☐

If No, describe what was done?

____

____

____

4. Was the study participant able to carefully remove the buffer cap?

Yes ☐

No ☐

If No, describe what was done?

____

____

____

5. Did the study participant correctly insert buffer cap into test stand?

Yes ☐

No ☐

If No, describe what was done?

____

____

____

6a. Was the study participant able to open disinfectant wipe?

Yes ☐

No ☐

If No, describe what was done?

____

____

____

6b. Was the study participant able to open sterile pad?

Yes ☐

No ☐

If No, describe what was done?

____

____

____

7. Did the study participant swab finger with disinfectant wipe and allow to dry?

Yes ☐

No ☐

If No, describe what was done?

____

____

____

8a. Did the study participant successfully uncap safety lancet?

Yes ☐

No ☐

If No, describe what was done?

____

____

____

8b. Did the study participant successfully place red end of lancet against the side of fingertip?

Yes ☐

No ☐

If No, describe what was done?

____

____

____

9. Did the study participant successfully press down firmly to prick their skin?

Yes ☐

No ☐

If No, describe what was done?

____

____

____

10. Did the study participant gently squeeze out the first blood drop?

Yes ☐

No ☐

If No then, describe what was done?

____

____

____

11. Did the study participant use sterile pad to wipe up blood?
    Yes ☐
    No ☐
    If No, describe what was done?
    ____
    ____
    ____

12. Did the study participant gently squeeze out second blood drop?
    Yes ☐
    No ☐
    If No, describe what was done?
    ____
    ____
    ____

13. Was the study participant able to fill the testing device with an adequate amount of blood?
    Yes ☐
    No ☐
    If No, describe what was done?
    ____
    ____
    ____

14a. Was the study participant able to position test device vertically above test stand?

    Yes ☐
    No ☐
    If No, describe what was done?
    ____
    ____
    ____

14b. Was the study participant able to push firmly through the foil cap until 3 snaps were felt?

    Yes ☐
    No ☐
    If No, describe what was done?
    ____
    ____
    ____

15. Was the study participant able to check for pink stain forming within 1 minute of puncturing the buffer pot?
    Yes ☐
    ☐

No
If No, describe what was done?

____
____

Process END time: ____ ____ : ____ ____

16. Did the participant quit the process at any point? If Yes, explain
____
____

17. Did the participant continue the process despite a missed or incorrect step?
____
____

Any other observer comments:
NOTES: (Was there significant hesitation or indecision at specific steps or overall? Did they say anything; did they ask any questions of the interviewer during the process? Which steps were skipped or modified?)
____
____
____
____

Section B. Result Interpretation

1. What did the participant interpret the following results as? Is the **control** line present?

Negative / Non-reactive

Negative ☐
Positive ☐
Invalid/test did not work ☐
Do not know/Not sure ☐
Other____

Faint positive / Low-reactive

Negative ☐
☐
☐
☐

Positive
Invalid/test did not work
Do not know/Not sure
Other____

Positive / Reactive

Negative ☐
Positive ☐
Invalid/test did not work ☐
Do not know/Not sure ☐
Other____

Invalid

Negative ☐
Positive ☐
Invalid/test did not work ☐
Do not know/Not sure ☐
Other____

Additional Interpretation Comments:

____
____
____

Section C. Post-test Interview

1. When was the last time you had an HIV test done?

   ____

2. Did you use the instructions sheet? ____

   If NO, please explain

   ____
   ____
   ____

3. Were the instructions easy to follow? Were the pictures helpful?

____
____
____

4. Was the device easy to use? ____
   If NO, please explain the steps that were difficult or confusing

____
____
____

5. How long should you wait before reading your result? __________________________

6. Were you confident with performing this test on your own?______________________
   If NO, please explain the steps that were difficult or confusing

____
____
____

7. Was it easy for you to INTERPRET the mock results? If NO, please explain (*Interviewer indicate which mock device they are referring to)*

____
____
____

8. What should you do if you have a negative result?

____
____
____

9. What should you do if you have a positive result?

____
____
____

10. What should you do if your test did not work?

____
____
____

11. How likely are you to use this test again?
    (Please tick one of the boxes below)

I will not use it	Unlikely	Unsure	Likely	Definitely use it

12. Would you prefer to use this test at home or get tested at a clinic?
(Please tick one of the boxes below)

Home	Clinic	Neither

13. How likely are you to recommend this test to a sexual partner, friend, or family member?
(Please tick one of the boxes below)

I will not recommend it	Unlikely	Unsure	Likely	Definitely recommend it

14. What are your favourite things about this test?
____
____
____

15. What are your least favourite things about this test?
____
____
____

HSTAR
HIV Self Testing Assessments & Research

HSTAR001 Data Collection Form

Section A. Test Performance

**Device type: Insti (Biolytical)**			**Researcher:**		**Date:**	
**Participant code:** ___ ___ ___ - ___ ___ ___ ___ - ___						
**Age:**	18-25 ☐	26-35 ☐	36-45 ☐	46-55 ☐	56-65 ☐	>65 ☐
**Dominant hand:**	Right ☐	Left ☐	**Gender:**	Male ☐	Female ☐	
**Education level:**	☐ ≤Grade 7 primary schooling level		☐ ≥Grade 8 primary schooling to ≤matric level		☐ ≥Technikon, University and University plus	

Process START time: ____ ____ : ____ ____

1. Did the study participant read/use the information sheet?
   Yes ☐
   No ☐

   If yes, was the IFU read before the test?

   Yes ☐
   No ☐

   Was it referred to during the test process?

   Yes ☐
   No ☐

   ____
   ____
   ____

2. Was it difficult for the study participant to remove the test device from the pouch?
   Yes ☐
   No ☐
   If Yes then, what seemed to be the problem? (E.g. could not find tear point, weakness, confusion)

   ____
   ____
   ____

3. Was the study participant able to remove the cap of Bottle 1?

Yes ☐
No ☐
If No, describe what seemed to be the problem?
___
___
___

4. Did the study participant twist the tip of the lancet off?

Yes ☐
No ☐
If No, describe what seemed to be the problem?
___
___
___

5. Did the study participant rub his/her finger correctly (up and down/vertical motion)?

Yes ☐
No ☐
If No, describe what seemed to be the problem?
___
___
___

6. Was the study participant able to lance his/her finger correctly?

Yes ☐
No ☐
If No, describe what seemed to be the problem?
___
___
___

7. Was the study participant able to form a blood droplet?

Yes ☐
No ☐
If No then, describe what seemed to be the problem?
___
___
___

8. Was the study participant able to get the blood droplet to fall into Bottle 1?
   Yes ☐
   No ☐
   If No then, how was it done?
   ____
   ____
   ____

9. Was the study participant able to twist the cap onto Bottle 1?
   Yes ☐
   No ☐
   If No then, how was it done?
   ____
   ____
   ____

10. Did the study participant shake Bottle1, 4 times?
   Yes ☐
   No ☐
   If No then, how many times ☐
   ____
   ____
   ____

11. Did the study participant pour the liquid from Bottle 1 into device and wait until liquid disappeared?
   Yes ☐
   No ☐
   If No then, how was it done?
   ____
   ____
   ____

12. Did the study participant shake Bottle 2, 4 times?
   Yes ☐
   No ☐
   If No then, how many times ☐
   ____
   ____
   ____

13. Did the study participant pour the liquid from Bottle 2 into device and wait until liquid disappeared?

    Yes ☐
    No ☐
    If No then, how was it done?

    ____
    ____
    ____

14. Did the study participant shake Bottle 3, 4 times?
    Yes ☐
    No ☐

    If No then, how many times ☐

    ____
    ____
    ____

15. Did the study participant pour the liquid from Bottle 3 into device and wait until liquid disappeared?
    Yes ☐
    No ☐
    If No then, how was it done?

____
____
____

Process END time: ____ ____ : ____ ____

16. Did the participant quit the process at any point? If Yes, explain

    ____
    ____

17. Did the participant continue the process despite a missed or incorrect step?

    ____
    ____

Any other observer comments:
NOTES: (Was there significant hesitation or indecision at specific steps or overall? Did they say anything; did they ask any questions of the interviewer during the process? Which steps

were skipped or modified?)

____
____
____
____

Section B. Result Interpretation

1. What did the participant interpret the following results as?

Negative / Non-reactive

Negative ☐
Positive ☐
Invalid/test did not work ☐
Do not know/Not sure ☐
Other____

Faint positive / Low-reactive

Negative ☐
Positive ☐
Invalid/test did not work ☐
Do not know/Not sure ☐
Other____

Positive / Reactive

Negative ☐
Positive ☐
Invalid/test did not work ☐
Do not know/Not sure ☐
Other____

Invalid

Negative ☐
Positive ☐
Invalid/test did not work ☐
Do not know/Not sure ☐
Other____

Additional Interpretation Comments:

____
____
____

Section C. Post-test Interview

1. When was the last time you had an HIV test done?
____
2. Did you use the Instructions sheet? ____
   If NO, please explain

3. Were the instructions easy to follow? Were the pictures helpful?

4. Was the device easy to use? ____
   If NO, please explain the steps that were difficult or confusing

5. How long should you wait before reading your result? ______________________________

6. Were you confident with performing this test on your own? ____________________
   If NO, please explain why you were not?

7. Was it easy for you to INTERPRET the mock results? If NO, please explain (*Interviewer indicate which mock device they are referring to*)

8. What should you do if you have a negative result?

9. What should you do if you have a positive result?

10. How likely are you to use this test again?
(Please tick one of the boxes below)

I will not use it	Unlikely	Unsure	Likely	Definitely use it

11. Would you prefer to use this test at home or get tested at a clinic?
(Please tick one of the boxes below)

Home	Clinic	Neither

12. How likely are you to recommend this test to a sexual partner, friend, or family member?
(Please tick one of the boxes below)

I will not recommend it	Unlikely	Unsure	Likely	Definitely recommend it

13. What are your favourite things about this test?

____

____

____

14. What are your least favourite things about this test?

____

____

____

HSTAR001 Data Collection Form

Device type: **Orasure**			Researcher:		Date:	
**Participant code:** ___ ___ ___ - ___ ___ ___ ___ - ___						
**Age:**	18-25 ☐	26-35 ☐	36-45 ☐	46-55 ☐	56-65 ☐	>65 ☐
**Dominant hand:**	Right ☐	Left ☐	**Gender:**	Male ☐	Female ☐	
**Education level:**	☐ ≤Grade 7 primary schooling level		☐ ≥Grade 8 primary schooling to ≤matric level		☐ ≥Technikon, University and University plus	

Section A. Test Performance

Process START time: ____ ____ : ____ ____

1. Did the study participant read/use the information sheet?

   Yes ☐
   No ☐

   If yes, was the IFU read before the test?

   Yes ☐
   No ☐

   Was it referred to during the test process?

   Yes ☐
   No ☐

   ____
   ____
   ____

2. Was it difficult for the study participant to remove the test tube from the pouch?

   Yes ☐
   No ☐

   If yes then, what seemed to be the problem? (e.g. couldn't find opening, weakness, could not find tear point)

   ____
   ____
   ____

3. Did the study participant remove the cap from the test tube?

   ☐
   ☐

Yes
No
If No then, why?

____
____
____

4. Did the study participant have difficulty with sliding the test tube into the stand?

Yes ☐
No ☐
If Yes then, why?

____
____
____

5. Was the study participant able to remove the test device from the pouch?

Yes ☐
No ☐
If No then, what seemed to be the problem?

____
____
____

6. Did the study participant touch the flat pad?

Yes ☐
No ☐
If Yes then, how was it done?

____
____
____

7. Did the study participant collect the sample correctly (1x upper and 1x lower swab)?

Yes ☐
No ☐
If No then, what did they do?

____
____
____

8. Did the study participant place the test device in the test tube correctly?

Yes ☐
No ☐
If No then, how was it done?
____
____
____

Process END time: ____ ____ : ____ ____

9. Which language was preferred by the user?
____

10. Did the participant quit the process at any point? If Yes, explain
____
____

11. Did the participant continue the process despite a missed or incorrect step?
____
____

Any other observer comments:
NOTES: (Was there significant hesitation or indecision at specific steps or overall? Did they say anything; did they ask any questions of the interviewer during the process? Which steps were skipped or modified?)
____
____
____
____

Section B. Result Interpretation

1. What did the participant interpret the following results as?

Negative / Non-reactive

Negative ☐
Positive ☐
Invalid/test did not work ☐
Do not know/Not sure ☐
Other____

Faint positive / Low-reactive

Negative ☐
Positive ☐
Invalid/test did not work ☐
Do not know/Not sure ☐
Other____

Positive / Reactive

Negative ☐
Positive ☐
Invalid/test did not work ☐
Do not know/Not sure ☐
Other____

Invalid

Negative ☐
Positive ☐
Invalid/test did not work ☐
Do not know/Not sure ☐
Other____

Additional Interpretation Comments:

____
____
____

Section C. Post-test Interview

1. Did you use the Instructions sheet? ______________________________________
   If NO, please explain

____
____
____

2. Were the instructions easy to follow? Were the pictures helpful?

____
____
____

3. Was the device easy to use? ____
   If NO, please explain the steps that were difficult or confusing

4. Were you confident with performing this test on your own? ____________________
   If NO, please explain why you were not?

5. Was it easy for you to INTERPRET the mock results? If NO, please explain (*Interviewer indicate which mock device they are referring to*)

6. What should you do if you have a negative result?

7. What should you do if you have a positive result?

8. What should you do if you have an invalid result?

9. What should you do if you are not sure of your result?

10. Would you use this test again?

11. Would you prefer to use this test at home or get tested at a clinic?

12. Would you recommend this test to a sexual partner/friend?

13. Do you have suggestions on how to make this product easier to use?

____

____

____
